# Impacts of aquaculture wastewater irrigation on soil microbial functional diversity and community structure in arid regions

**DOI:** 10.1038/s41598-017-11678-z

**Published:** 2017-09-11

**Authors:** Lijuan Chen, Qi Feng, Changsheng Li, Yongping Wei, Yan Zhao, Yongjiu Feng, Hang Zheng, Fengrui Li, Huiya Li

**Affiliations:** 1 Key Laboratory of Ecohydrology of Inland River Basin, Northwest Institute of Eco-Environment and Resources, Chinese Academy of Sciences, Lanzhou, 730000 China; 2 School of Earth and Environmental Sciences, The University of Queensland, Brisbane, 4072 Australia; 3 Plant Protection and Quarantine Station of Gansu Province, Lanzhou, 730020 China; 4 College of Marine Sciences, Shanghai Ocean University, Shanghai, 201306 China; 5 School of Environment and Civil Engineering, Dongguan University of Technology, Dongguan, Guangdong 523106 China

## Abstract

Aquaculture wastewater is one of the most important alternative water resources in arid regions where scarcity of fresh water is common. Irrigation with this kind of water may affect soil microbial functional diversity and community structure as changes of soil environment would be significant. Here, we conducted a field sampling to investigate these effects using Biolog and metagenomic methods. The results demonstrated that irrigation with aquaculture wastewater could dramatically reduce soil microbial functional diversity. The values of diversity indices and sole carbon source utilization were all significantly decreased. Increased soil salinity, especially Cl concentration, appeared primarily associated with the decreases. Differently, higher bacterial community diversity was obtained in aquaculture wastewater irrigated soils. More abundant phyla Actinobacteria, Chloroflexi, Acidobacteria, Gemmatimonadetes and fewer members of Proteobacteria, Bacteroidetes and Planctomycetes were found in this kind of soils. Changes in the concentration of soil Cl mainly accounted for the shifts of bacterial community composition. This research can improve our understanding of how aquaculture wastewater irrigation changes soil microbial process and as a result, be useful to manage soil and wastewater resources in arid regions.

## Introduction

As the world needs about 60% more food to feed the 9 billion people in 2050^1^, sustainable management and judicious use of land and water resources appears extremely vital^2^. This is especially important in arid and semiarid regions where commonly scarcity of fresh water forces farmers to use water from other sources to cultivate thirsty crops^3^. Wastewater is often proposed as one of these most important water resources. Irrigation with this kind of water could not only significantly relieve pressure on fresh water resources, but also alleviate the discharge of effluents into water environment, avoiding in this way the deterioration of fresh water ecosystems associated with eutrophication and algal bloom^4^. The use of wastewater in irrigation has also been found to have additional agronomic benefits associated with soil structure and fertility. According to Kiziloglu *et al*.^5^, wastewater has a high nutritive value that may reduce fertilizer application rates and increase productivity of poor fertility soils. Diverse studies have indeed shown that wastewater irrigation increases soil organic matter^6, 7^. However, potential risk follows as a shadow aspect. Detrimental effects of wastewater irrigation on soil quality, such as increase salinity and decrease soil pH^8, 9^, as well as increase soil heavy metal contamination^10–12^, have been reported as a research topic for decades. Meanwhile, irrigation with wastewater is also associated with several biological risks, i.e., the presence of pathogens, viruses, helminthes and protozoa in soils^13–15^, which have opened a new controversial front in the public debate^16^.

Soil microbes are one of the most complex components of soil ecosystem^17^. They are not only the principal participants and drivers of biogeochemical cycling of elements^18^, but also the sensitive indicators of soil environmental change^19^. Hidri *et al*.^20^ found that long-term irrigation with treated wastewater resulted in increased soil microbial abundance and induced in particular compositions of the bacterial and fungal communities. Oved *et al*.^21^ and Ndour *et al*.^22^ investigated that wastewater irrigation produced shifts in ammonia-oxidizing bacteria population in soils, as compared to soils irrigated with freshwater. However, no difference was found by Ndour *et al*.^22^ between the two treatments in microbial biomass or microbial activities (measured as fluorescein diacetate activity). Truu *et al*.^23^ reported that it was the willow growth rather than wastewater irrigation affected soil microbiological and biochemical properties under short term municipal wastewater irrigation. As described by Lopes *et al*.^24^, the direct microbiological risks associated with the use of wastewater in agricultural irrigation were mainly the disturbance of the indigenous microbial communities in soils and the influence on their functional activities. Nevertheless, our understanding of these aspects has much room for improving.

Generally, the compound and concentration of aquaculture wastewater are different from municipal and industrial wastewater, which are expected to stimulate different organisms and metabolic pathways^25^. Several studies have focused on the effects of aquaculture wastewater irrigation on soil chemical and physical processes^26, 27^. To the best of our knowledge, however, up to now few studies assess the comprehensive effects on soil microbial functional characteristics and bacterial community composition due to long-term use of aquaculture wastewater irrigation. In addition, as the next generation sequencing methods have commonly used for mapping soil microbial phylogeny^28^, it is surprising that little research about the effects of aquaculture wastewater irrigation on soil microbes is involving in this. With this review, we conducted samplings in fresh and aquaculture wastewater irrigated grape fields in a typical arid region to investigate the differences of microbial functional diversity and bacterial community structures in two soils and analyze the relationships of microbial characteristics and the abiotic environment. The main objective of this study is to find out the effects of aquaculture wastewater irrigation on soil microbial properties and analyze the possible reasons. We hope this research can contribute to generate the progress in the understanding of how aquaculture wastewater irrigation changes the structure and function of soil ecosystem by influencing soil microbial process and as a result, be useful to manage soil and wastewater resources in arid regions.

## Results

### Microbial functional diversity

**Figure 1:**
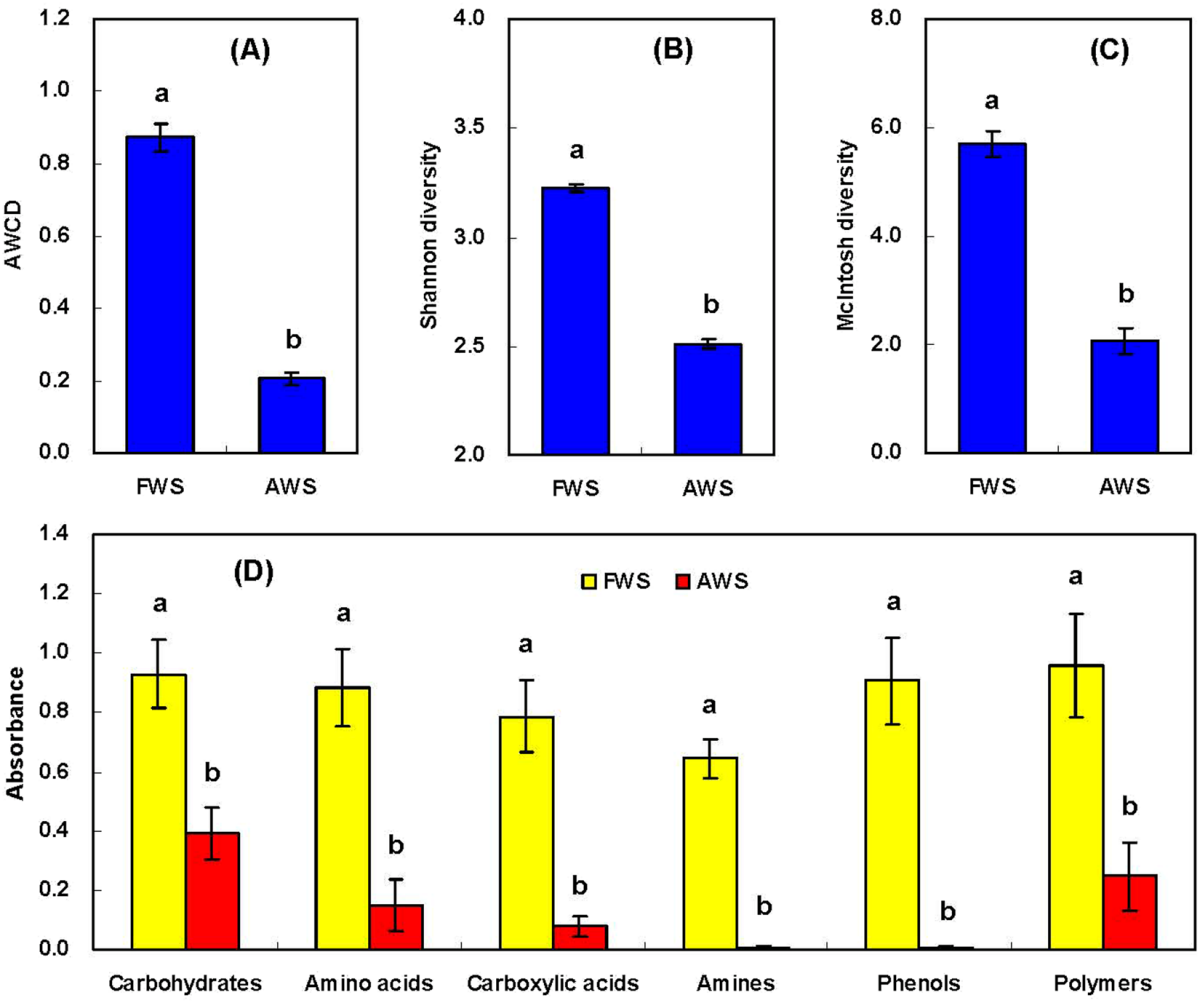
Effects of aquaculture wastewater irrigation on microbial functional diversity indices (**A**–**C**) and utilization of six functional categories of carbon substrates (**D**). Values with different letters indicated significant difference at *P* < 0.05 according to the paired *t*-test. FWS: fresh water irrigated soils; AWS: aquaculture wastewater irrigated soils.

**Figure 2:**
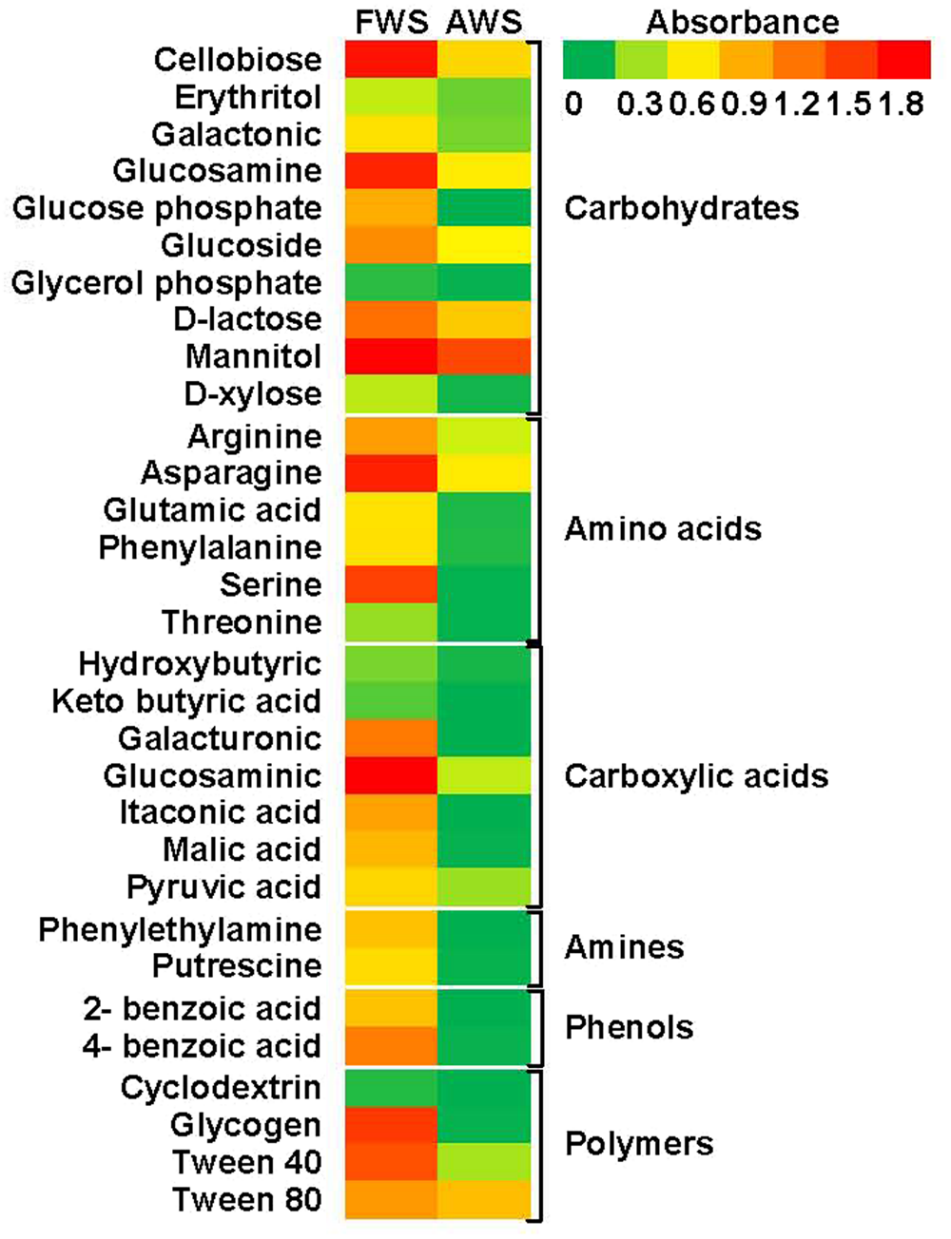
Heatmap of 31 carbon substrates utilization under different treatments. FWS: fresh water irrigated soils; AWS: aquaculture wastewater irrigated soils.

Remarkable differences in microbial functional characteristics were detected in fresh (FWS) and aquaculture wastewater irrigated soils (AWS). The values of average well color development (AWCD), which represented the metabolic activities of soil microbial communities in using carbon sources, were significantly lower in AWS versus FWS (*P* < 0.05; Fig. 1A). Meanwhile, there was a significant metabolic diversity reduction in AWS, as shown by the values of Shannon and McIntosh diversity indices (Fig. 1B and C). This trend continued in carbon substrates consumption. The utilizations of six functional categories of carbon substrates were all significantly reduced in AWS (Fig. 1D). Especially, amines and phenols were difficultly consumed by microbes, which were also confirmed from the scarcity in Phenylethylamine, Putrescine, 2- benzoic acid and 4- benzoic acid uptake (Fig. 2). Although the carbohydrates (44.8%) and polymers (28.1%) were the dominant categories consumed by microbes in AWS, the consumption of each sole carbon source was unexceptionally significantly decreased compared in FWS (*P* < 0.05). Under this circumstance, although totally 24 sole carbon sources could be used in AWS, mainly 7 sources had higher values, i.e. cellobiose, glucosamine, glucoside, d-lactose, mannitol, asparagine and tween 80, which accounted for 9.3, 7.5, 6.8, 10.5, 21.8, 7.7 and 11.5% of total substrates consumption respectively.

**Figure 3:**
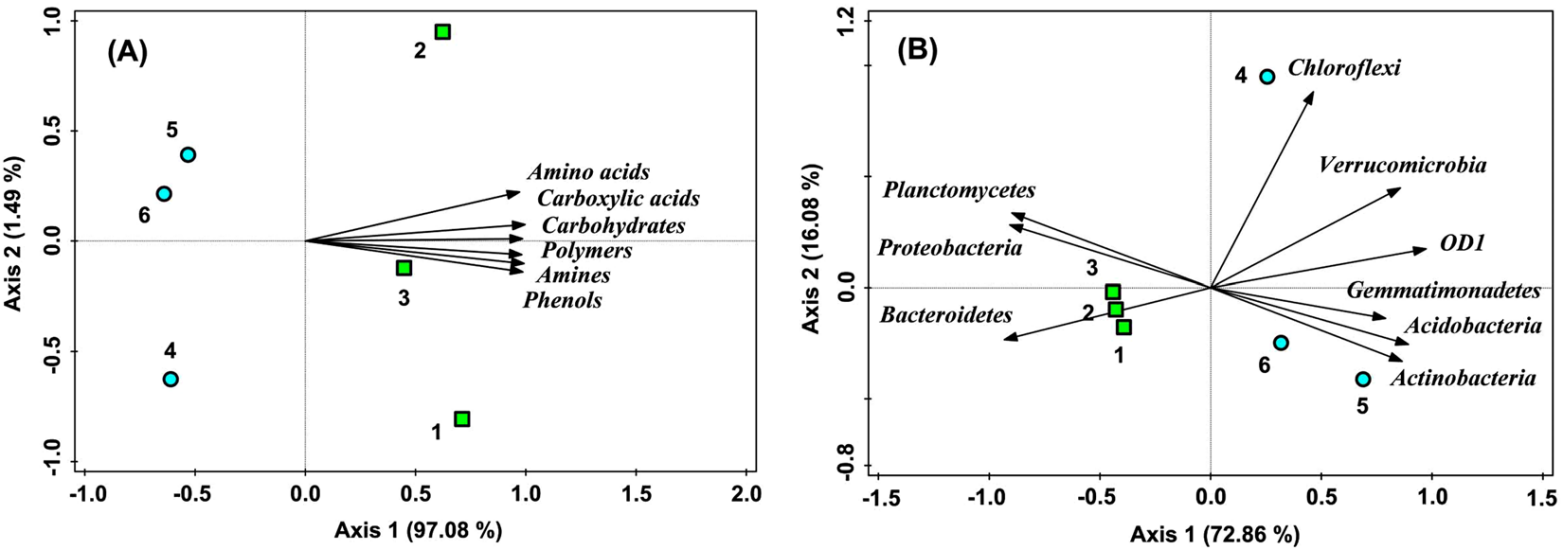
Principal component analysis (PCA) of the utilization of six functional categories of carbon substrates (**A**) and relative abundances of bacterial phyla (**B**). Vectors in black color represent microbial characteristics. Green squares and blue circles with numbers indicate samples from fresh (FWS) and aquaculture wastewater irrigated soils (AWS).

Principal component analysis (PCA) showed that the samples 1, 2 and 3 in FWS (with green color) and 4, 5 and 6 in AWS (with blue color) were clearly separated along the Axis 1 (Fig. 3A). Meanwhile, three samples in FWS (on the right) were more scattered than in AWS (on the left). For example, microbes in FWS sample 2 more liked to consume amino acids while phenols, amines and polymers were more preferred by FWS-sample-1 microbes; soil microbes in FWS sample 3 consumed the least carbon substrates among them, demonstrating the minimum microbial metabolic activities in it. Differently, samples 5 and 6 in AWS almost grouped together, indicting that the microbes in them had similar metabolic activities. Based on the PERMANOVA analysis, the significant differentiation of aquaculture wastewater irrigation on microbial carbon sources utilization patterns (*F* = 71.59, *P* < 0.001) was confirmed. All six carbon categories consumed by soil microbes showed strong positive correlations with the Axis 1, demonstrating that they were together determined the array of the samples.

### Composition of the bacterial communities

**Figure 4:**
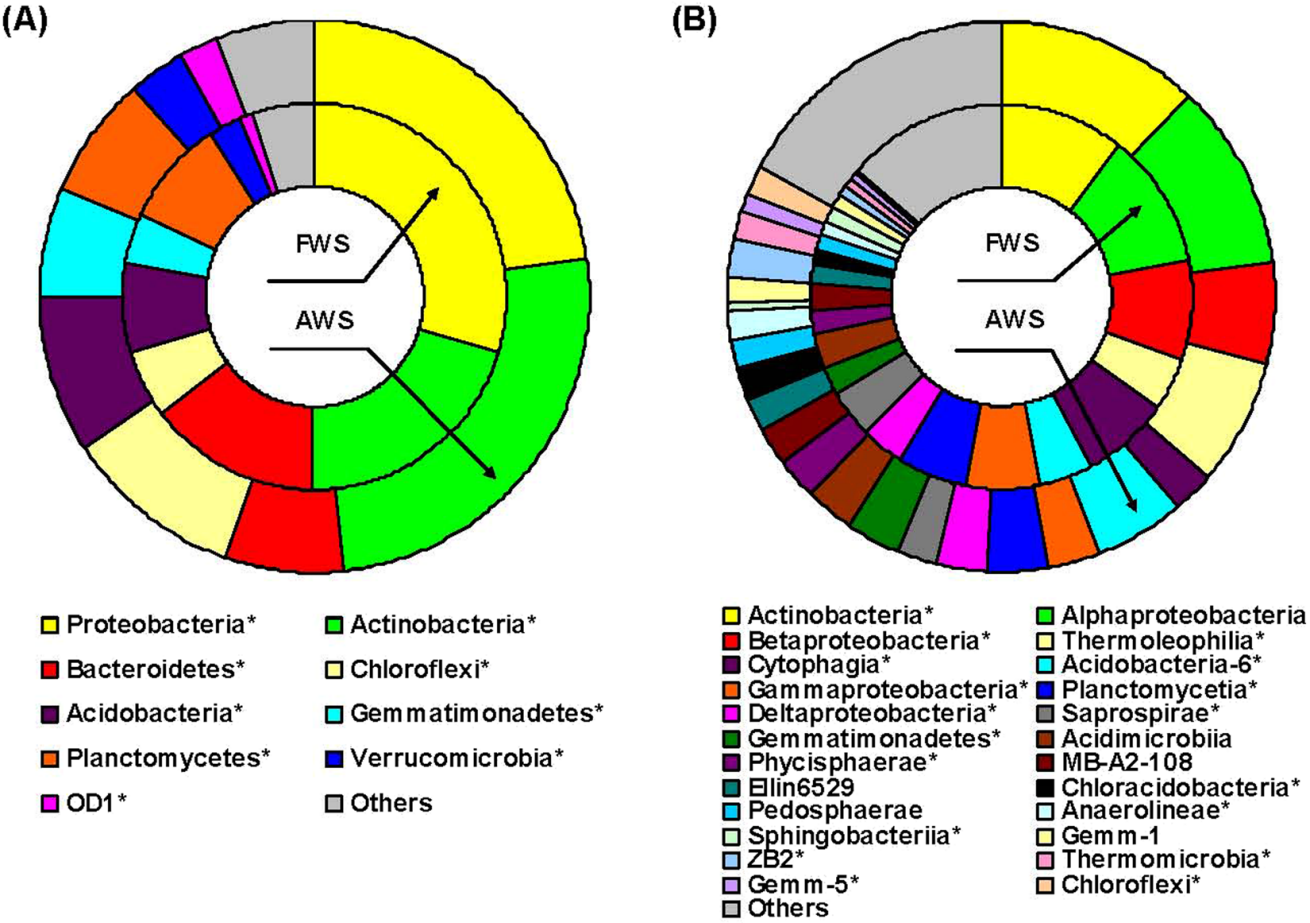
Rings represent the average relative abundance (from three replicate samples) of bacterial phyla that made up at least 1% of the whole community (**A**) and classes that made up at least 0.2% of the whole community (**B**); values that were significantly different in relative abundance between fresh (FWS) and aquaculture wastewater irrigated soils (AWS) are marked by asterisks (P < 0.05, paired t test).

Aquaculture wastewater irrigation could also significantly change the bacterial community structures. The number of bacterial operational taxonomic units (OTUs) and the values of community diversity indices (Chao 1 estimator, abundance-based coverage estimator (ACE), Shannon and Simpson) in AWS were all significantly higher than in FWS (*P* < 0.05, Table 1). Proteobacteria, Actinobacteria, Bacteroidetes, Chloroflexi, Acidobacteria, Gemmatimonadetes, Planctomycetes, Verrucomicrobia and OD1 were the dominant bacterial phyla in both FWS and AWS (Fig. 4A). The relative abundance of Proteobacteria, Bacteroidetes and Planctomycetes significantly reduced from FWS to AWS, the values of which shifted from 29.7 to 22.9%, 14.4 to 6.9% and 8.7 to 7.3%, respectively (*P* < 0.01). Figure 4B showed that the main drivers for this reduction at the class level were Betaproteobacteria and Gammaproteobacteria (phylum of Proteobacteria), Cytophagia (Bacteroidetes) and Planctomycetia (Planctomycetes), with the relative abundance reduced from 8.2 in FWS to 5.8% in AWS, 6.4 to 3.0%, 7.6 to 2.8% and 5.8 to 3.6%, respectively. Differently, phyla Actinobacteria (20.5 in FWS to 25.5% in AWS), Chloroflexi (5.8 to 10.6%), Acidobacteria (7.3 to 9.1%) and Gemmatimonadetes (4.3 to 6.5%) were the dominant increased members in AWS (*P* < 0.01), with classes Actinobacteria and Thermoleophilia in phylum of Actinobacteria increased from 10.6 to 12.0% and 4.3 to 7.4% respectively. Some other classes, albeit the relative abundances were not dramatic, such as Phycisphaerae, Chloracidobacteria and ZB2, were also found richer in AWS.

**Table 1:** Means and standard deviation of bacterial community richness and diversity indices across irrigation water salinity gradient.

Treatment	OTUs	Chao 1	ACE	Shannon
FWS	3399 (362) a	9472 (298) a	9630 (235) a	7.38 (0.013) a
AWS	3612 (301) b	15047 (456) b	17086 (322) b	7.60 (0.014) b

Values with different letters indicated significant difference at *P* < 0.05 according to the paired *t*-test. FWS: fresh water irrigated soils; AWS: aquaculture wastewater irrigated soils; OTUs: operational taxonomic units; ACE: abundance-based coverage estimator.

PCA ordination showed that the bacterial communities in FWS (with green color on the left) were less varied than in AWS (with blue color on the right) as the three samples in AWS were more scattered (Fig. 3B). Along the Axis 1, samples 4, 5 and 6 in AWS mainly located at the positive direction of the Axis 1 while samples 1, 2 and 3 in FWS chiefly at the negative direction. PERMANOVA analysis also showed significant differentiation of bacterial composition at the phylum level in FWS and AWS (*F* = 7.30, *P* < 0.001). Phyla OD1, Acidobacteria, Verrucomicrobia, Actinobacteria and Gemmatimonadetes which were more dominant in AWS (Fig. 4A) showed strong positive loadings in comparison to Bacteroidetes, Proteobacteria and Planctomycetes (more rich in FWS) with strong negative loadings. Phylum Chloroflexi which had high abundance in AWS sample 4 showed strong positive correlation with Axis 2, revealing that Chloroflexi was the decisive phylum to distinguish sample 4 from 5 and 6.

### Microbial characteristics shifts versus soil environmental variables

**Figure 5:**
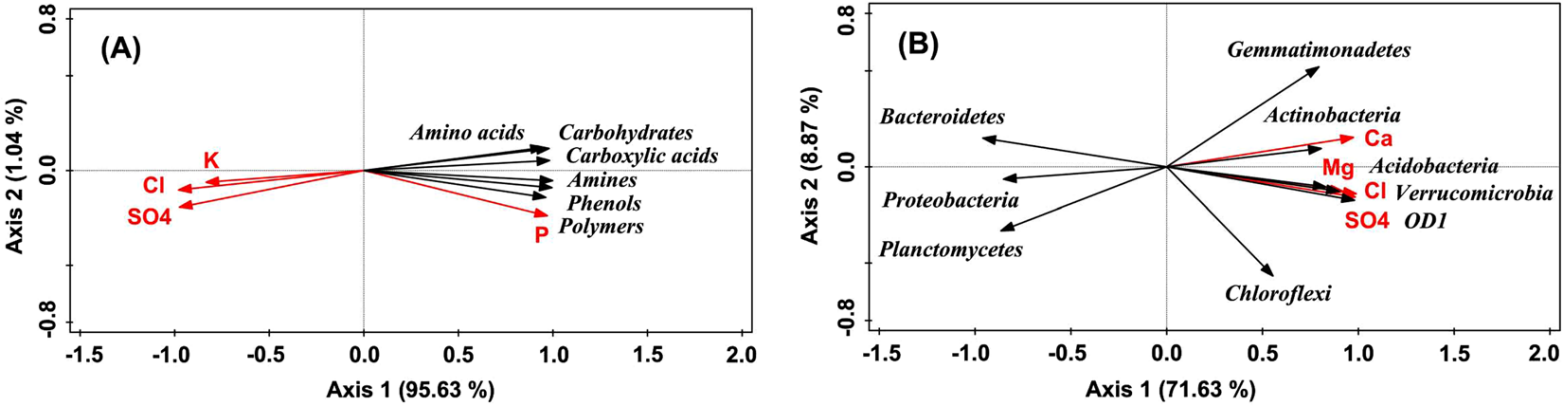
Redundancy analysis (RDA) of the utilization of six functional categories of carbon substrates (**A**) and relative abundances of bacterial phyla (**B**) constrained by soil chemical properties. Vectors in red color represent selected soil chemical properties and in black color represent microbial characteristics. Before the RDA, selection of the soil variables using the stepwise regression method and the Monte Carlo Permutation test was conducted.

Soil environment changes which induced by aquaculture wastewater irrigation could significantly influence microbial functional characteristics and community structures. High concentration of soil total phosphorus (P) favored the increase in the consumption of Carbohydrates, Carboxylic acids, Amines, Phenols and Polymers (Table 2). The concentrations of K, Cl and SO_4_ were significantly and negatively correlated with all carbon substrates' utilization (*P* < 0.01). Meanwhile, the most difficultly consumed carbon sources, amines and phenols, in AWS were negatively affected by soil Na, Ca and Mg concentrations, demonstrating that these salt ions played an important role in the consumption of these carbon sources. The Axis 1 of redundancy analysis (RDA) showed a clear environmental gradient of increasing P and decreasing K, Cl and SO_4_, which separated all carbon substrates utilization to the positive direction of the Axis 1 (Fig. 5A). Although these most effective soil environmental variables, i.e. P, K, Cl and SO_4_, explained 99.2% of the overall variation in the substrates consumption, only soil Cl concentration were significant in the Monte Carlo permutation test (*F* = 58.1, *P* = 0.018).

**Table 2:** Spearman correlations between soil microbial characteristics and soil environmental variables.

	pH	C	N	P	EC	K	Na	Ca	Mg	Cl	SO_4_	HCO_3_	NO_3_
**Utilization of six functional categories of carbon substrates**													
Carbohydrates	0.51	0.48	−0.13	**0.92**	−0.79	−**0.96**	−**0.92**	−0.91	−**0.92**	−**0.98**	−**0.98**	0.78	−0.83
Amino acids	0.73	0.48	−0.20	0.89	−0.62	−**0.93**	−0.83	−0.79	−0.84	−**0.93**	−**0.94**	0.85	−0.67
Carboxylic acids	0.62	0.41	−0.24	**0.94**	−0.70	−**0.97**	−0.91	−0.87	−0.91	−**0.97**	−**0.97**	0.80	−0.78
Amines	0.63	0.34	−0.34	**0.98**	−0.68	−**0.94**	−**0.92**	−**0.93**	−**0.96**	−**0.97**	−**0.96**	0.69	−0.80
Phenols	0.64	0.33	−0.36	**0.99**	−0.66	−**0.93**	−**0.93**	−**0.93**	−**0.95**	−**0.96**	−**0.95**	0.67	−0.79
Polymers	0.58	0.32	−0.35	**0.97**	−0.60	−**0.98**	−**0.97**	−0.84	−0.89	−**0.93**	−**0.91**	0.78	−0.73
**Main phyla of bacterial community**													
Proteobacteria	0.14	0.70	0.36	0.63	−**0.93**	−0.72	−0.70	−0.80	−0.70	−0.84	−0.81	0.68	−0.80
Bacteroidetes	0.65	0.44	−0.25	**0.96**	−0.70	−**0.93**	−0.90	−0.91	−**0.93**	−**0.97**	−**0.97**	0.75	−0.78
Actinobacteria	0.06	−0.16	0.09	−0.73	0.82	0.77	0.86	0.86	0.82	0.77	0.81	−0.41	0.72
Acidobacteria	−0.14	−0.65	−0.28	−0.67	0.87	0.80	0.78	0.77	0.70	0.84	0.83	−0.78	0.77
Chloroflexi	−0.79	−0.04	0.42	−0.59	0.26	0.64	0.46	0.45	0.59	0.63	0.63	−0.51	0.38
Gemmatimonadetes	−0.25	−0.49	−0.19	−0.54	0.83	0.43	0.43	0.81	0.70	0.72	0.68	−0.22	0.76
Planctomycetes	0.08	0.27	−0.09	0.77	−0.83	−0.71	−0.82	−**0.93**	−0.86	−0.80	−0.82	0.35	−0.91
Verrucomicrobia	−0.55	−0.19	0.25	−0.78	0.75	0.79	0.70	0.87	0.91	0.91	0.91	−0.47	0.85
OD1	−0.51	−0.38	0.19	**−0.93**	0.80	**0.92**	**0.92**	**0.94**	**0.95**	**0.99**	**0.99**	−0.72	0.88

Values at *P* < 0.01 are shown in bold. C: organic carbon; N: total nitrogen; P: total phosphorus; EC: electrical conductivity.

The phyla which were more sensitive to soil environment changes were Bacteroidetes and OD1 under the circumstance of aquaculture wastewater irrigation (Table 2). However, absolutely opposite influence was detected as the relative abundance of Bacteroidetes was correlated positively with soil P and negatively with soil K, Mg, Cl and SO_4_ concentrations while the abundance of OD1 was negatively correlated with soil P but positively with soil K, Na, Ca, Mg, Cl and SO_4_ concentrations. Phylum Proteobacteria was mainly affected by soil EC (electrical conductivity) although no exactly ions were involved and soil Ca concentration was negatively correlated with the abundance of Planctomycetes. RDA showed total 88.4% of the variation in bacterial community structures was explained (Fig. 5B), in which 70.3% was caused by Cl, 8.5% by Ca, 5.7% by SO_4_ and 3.9% by Mg. Soil Cl concentration was significant in the Monte Carlo permutation test with *F* = 9.5 and *P* = 0.01, suggesting that Cl was also the dominant influence factor of the soil bacterial community structures.

## Discussion

In some arid and semiarid regions, the use of wastewater in agriculture irrigation is crucial for overall water management^29, 30^ although it may alter soil environment greatly and as a result, affect soil microbes. In this study, total microbial metabolic activities and the values of functional diversity indices were detected significantly decreased in AWS. The utilization of six functional categories of carbon substrates was also significantly reduced. In addition, excepting of 7 sole carbon sources, 24 sources could not or merely little be used by soil microbes in AWS. PCA had displayed that the metabolic diversity in AWS was lower than in FWS. These results indicated that aquaculture wastewater irrigation have exerted adverse effects on soil microbial functional diversity. Formerly, Tam^31^ observed a similar decrease in microbial activities in mangrove soils which had been irrigated with saline wastewater; Brzezińska^32^ found irrigation with municipal wastewater reduced catalase activity at the high irrigation dose; Kayikcioglu^33^ reported a decrease of the activities of enzymes aryl sulfatase, dehydrogenase, urease, alkaline phosphatase and β-glucosidase in wastewater irrigated agricultural soils. However, Truu *et al*.^23^ reported a significant increase of alkaline phosphatase in soils irrigated with secondary-treated municipal wastewater over 2 years and Chen *et al*.^29^ observed an enhancement of various enzymatic activities in soils irrigated with reclaimed wastewater over 10 years. These differences may mainly come from the compounds of wastewater and their concentration, the duration of irrigation and the properties of the soil irrigated^24, 34^, which decided the final effects of wastewater irrigation on soil microbial functional characteristics.

Differently, the richness and diversity of bacterial communities were all significantly higher in AWS. PCA also showed that bacterial communities were clustered in FWS and random in AWS. This pattern is consistent with the result of Li *et al*.^35^, who found that wastewater irrigation significantly increased the bacterial diversity based on denaturing gradient gel electrophoresis (DGGE) analysis. As for the bacterial community composition, the relative abundances of Proteobacteria, Bacteroidetes and Planctomycetes reduced significantly from FWS to AWS while for that of Actinobacteria, Chloroflexi, Acidobacteria and Gemmatimonadetes increased. Generally, the Proteobacteria encompass an enormous level of morphological, physiological and metabolic diversity, and are of great importance to global carbon cycling^36^. The majority of Proteobacteria are thought to grow fast, preferring nutrient-rich environments and are shown to be positively correlated with soil CO_2_ production^37^. Indeed, the observed reduction was consistent with decreased organic carbon in aquaculture wastewater irrigated soil (from 1.38 in FWS to 1.25 g kg^−1^ in AWS). One possible reason for this result is a significant soil salinity increase under aquaculture wastewater irrigation^38^. Wu *et al*.^39^ showed that the relative abundance of the Betaproteobacteria decreased with increasing salinity, whereas that of the Alphaproteobacteria and the Gammaproteobacteria increased. However, in study of soils from the former lake Texcoco, Valenzuela-Encinas *et al*.^40^ showed that the dominant class of Proteobacteria in both high and low saline soils was Gammaproteobacteria, whereas in medium saline soils was Alphaproteobacteria. Different region and soil characteristics may contribute to these differences. In our study, although the abundances of both these three classes decreased in AWS, Betaproteobacteria and Gammaproteobacteria were the significant derivers for the Proteobacteria relative abundance reduction. The phyla Actinobacteria, Chloroflexi, Acidobacteria and Gemmatimonadetes were found the most abundant bacterial groups thriving in both wastewater and agricultural soil^25^. The relative abundance increases for these phyla may suggest that bacteria in wastewater could become part of the soil microbes through the use of irrigation. A following result may be the decrease of the other phyla induced by competition (such as Proteobacteria, Bacteroidetes and Planctomycetes). In addition, these effects of introduced microbes may be related not only with the interference that exogenous populations may have on the soil microbial community, but also with the capacity of the exogenous organisms to survive in soil and constitute a health risk to soil ecological system and quality^25^.

Based on the above results, we found that although the soils irrigated with aquaculture wastewater exhibited higher bacterial community richness and diversity (Table 1), they showed lower microbial functional diversity (Fig. 1A–C). This result indicates that aquaculture wastewater irrigated soil organisms are functionally weak even though they remain high taxonomic diversity. One possible explanation for this result is a shift from specialist species (harboring specialized functional genes) to generalist species (harboring functional genes shared by many species). Indeed, an increase in the taxonomic diversity of generalist species will not result in an increase in functional diversity, since most species harbor more or less the same genes while in contrast, an increase in the taxonomic diversity of specialist species will result in an increase in functional diversity, since each species harbors a specific set of functional genes^41^. Under this circumstance, the observed reduction of functional diversity in this study may attribute to the decreases in the taxonomic diversity of specialist species in Proteobacteria, Bacteroidetes and Planctomycetes while the increases in the Actinobacteria, Chloroflexi, Acidobacteria and Gemmatimonadetes could not increase the taxonomic diversity of specialist species. In addition, consequences of such shifts may also change some specialized soil functions. As Smalla *et al*.^42^ discovered, by comparing the temperature gradient gel electrophoresis (TGGE) profiles of the original inoculum with those in the BIOLOG wells following incubation, that fast-growing bacteria which adapted to high substrate concentrations were numerically dominant in the BIOLOG wells. Therefore, the comparatively high diversities of specialist species may more accurately reflect the functional community. In this study, however, as the patterns of substrate utilization only indicated functional aspects of the culturable fraction of the community inoculated to the wells, the shifts in bacterial community composition may be less well represented the functional changes, thus, future work would be needed for more accurate assessment.

According to the relationship analysis (Table 2 and Fig. 5), it appeared that aquaculture wastewater induced reduction in microbial functional properties were primarily associated with decreased P and increased soluble salts (mainly K, Cl and SO_4_) concentration in soils while the variation of bacterial community structures was mainly correlated with P, EC and ions (mainly Ca, Mg, Cl and SO_4_) concentration. Generally, aquaculture wastewater tend to be very saline (15 dS m^−1^ < EC < 45 dS m^−1^) and often have higher nutrient levels, such as phosphorus and nitrogen^38^. Although muds desorption (used in our sampling region) as a physical chemical water treatment technology was observed to be effective in removing phosphorus^43^, soluble salts left in wastewater would still inevitably promote soil salinisation by irrigation, which in fact was the most commonly reported negative effects of wastewater irrigation^44, 45^. The positive relationship between phosphorus and microbial functional diversity had been reported by many studies^46, 47^. As the soil total phosphorus decrease was indeed detected in our study (from 10.72 in FWS to 7.87 mg kg^−1^ in AWS), the reduction of soil microbial functional diversity and their significant correlation could be understood. Majority previous studies had concerned the negative impacts on soil microbial activities as soil salinity increased^48–50^. Jin *et al*.^51^ found AWCD values decreased significantly with increasing soil salinity. Setia *et al*.^49^ reported that saline soil (electrical conductivity bigger than 5.0 dS m^−1^) reduced soil respiration even by more than 50%. However, the effects of soil salinity or salt ions on microbial community structure are still fragmented and incomplete, especially based on metagenomic methods^52^. Our result demonstrated that bacterial phyla Bacteroidetes had completely different response to salt ions compared with OD1, although they were all sensitive to salt ions concentration changes. As the correlation analysis used in this study were just based on two treatments including 6 samples, more work should be done in future to clearly distinguish the response of different microbes to salt ions. Increased soil Cl concentration induced by aquaculture wastewater irrigation were detected playing a primarily role in the reduction of microbial functional diversity and the variation of bacterial communities in our study. It is not surprising as salinity toxicity is often attributed to the Cl that is massively present in saline soils^53, 54^. Zahran^55^ found that the microbial growth was significantly inhibited by the toxicity of Cl. Gryndler *et al*.^56^ also detected Cl concentration would mainly affect indigenous microorganism. Many biogeochemical processes would also be influenced by soil Cl concentration, such as nitrification, which was reported inhibited by chloride salts^57^. These results demonstrated that more attention should be paid to soil Cl concentration control in order to alleviate negative effects of aquaculture wastewater irrigation on soil ecosystem.

## Methods

### Site description and sampling

In order to undertake the present study, a cold-water fish breeding station, which is located in Yangguan Town, northwest China (94°01′33.7″E, 39°55′38.1″N) was chosen as the sampling region. As sit at the boundary of Kumtag Desert, the mean annual precipitation of this region is only 39.9 mm and the annual temperature is 9.3 °C. The station covered approximately 30,000 m^2^ areas and bred about 8,000 fish in 2014. Around the station, grapes were planted in more than 10 ha farmlands. A lake (locally called Moon Lake), the water source of which was Qilian glacial snowmelt, supplied water for fish breeding and the most of grape fields irrigation. The wastewater from all fish ponds were collected, muds-adsorption treated and transported into designed grape fields near the station. The chemical composition of lake fresh water and treated wastewater were shown in Table 3. Above 5 years irrigation application had been conducted.

**Table 3:** Chemical composition of irrigation water.

Water	K (mg L^−1^)	Na (mg L^−1^)	Ca (mg L^−1^)	Mg (mg L^−1^)	Cl (mg L^−1^)	SO_4_ (mg L^−1^)	HCO_3_ (mg L^−1^)	EC_w_ (dS m^−1^)
FW	7.16	122.47	27.00	66.54	147.68	307.08	82.37	0.71
AW	24.80	321.50	136.00	127.65	785.10	890.64	36.04	2.29

Mean value of three replicate samples. FW: fresh water; AW: aquaculture wastewater; EC_w_: electrical conductivity of water.

Soil samples were collected from two kinds of grape fields on July 16^th^, 2014. One kind was irrigated with water from Moon Lake (fresh water irrigated soil) and another with aquaculture wastewater. In each kind of field, 3 sites were chosen as duplicates of sampling. Totally 13 non-rhizosphere soil cores (0–20 cm depth) were collected from each site according to an *S*-shaped curve, and then completely mixed into one composite soil sample. After sieving out plant roots and stones, 1 kg of soil sample was obtained and put into a sterile bag. Totally 6 soil samples were obtained and packed in ice blocks and then transported to laboratory within 24 h, where each sample was divided into three parts. One part was refrigerated at 4 °C for microbial functional diversity analysis, a second part was frozen at −80 °C for microbial genetic analysis and others were air dried for chemical analysis.

### Soil chemical analysis

Soil organic carbon (C) was analyzed by colorimetry after oxidation with a mixture of potassium dichromate and sulphuric acid^58^. Total nitrogen (N) was measured using the Kjeldahl method^59^. Total phosphorus (P) was determined by NaHCO_3_ (0.5 M, pH 8.5) extraction^60^. The pH and EC were determined in a soil suspension with deionized water (1:5 w/v). Detailed salinity (K, Na, Ca, Mg, Cl, SO_4_, HCO_3_ and NO_3_) was measured by the ion exchange chromatography (ICS 5000, Dionex). The results were shown in Table 4.

**Table 4:** Soil chemical characteristics under different treatments.

Treatment	pH	C (g kg^−1^)	N (g kg^−1^)	P (mg kg^−1^)	EC (dS m^−1^)	K (mg kg^−1^)	Na (mg kg^−1^)	Ca (mg kg^−1^)	Mg (mg kg^−1^)	Cl (mg kg^−1^)	SO_4_ (mg kg^−1^)	HCO_3_ (mg kg^−1^)	NO_3_ (mg kg^−1^)
FWS	8.62	1.38	0.37	10.72	0.23	3.81	9.66	6.02	8.16	42.60	49.39	141.58	1.22
AWS	8.33	1.25	0.40	7.87	0.31	4.77	14.68	10.03	11.09	76.68	96.40	122.27	1.99

Mean value of three replicate samples. FWS: fresh water irrigated soils; AWS: aquaculture wastewater irrigated soils; C: organic carbon; N: total nitrogen; P: total phosphorus; EC: electrical conductivity.

### Microbial functional diversity analysis

Soil microbial metabolic activity was measured using Biolog Ecoplates^TM^. The plates have 96 wells and each plate consisting of three replicates (comprising 31 sole carbon sources and one water blank). In this study, 5 g of each soil sample was suspended in 45 ml of sterile saline solution (0.85% NaCl) and shaken 30 min on an orbital shaker. Then 1 ml of soil suspension was transferred into a microcentrifuge tube and centrifuged at 10,000 rpm for 20 min. The supernatant was removed. The pellets were washed twice to remove water soluble carbon using the sterile saline solution and resuspended in 20 ml of the same solution. A 150 μl sample of the suspension was inoculated into each well. The plates were incubated at 25 °C. Color development in each well was recorded as optical density at 595 nm and 750 nm at 24 h intervals for 168 h.

The well absorbance values were adjusted by subtracting the absorbance of the control well. The final values in each well were the 590 nm values minus the 750 nm values. Negative readings were set to zero^61^. Microbial activity in each microplate, expressed as AWCD value was determined according to Garland and Mills^62^. The 168 h optical density value (chosen according to exponential phase of growth curves of all plates) for each sample in triplicate, divided by their AWCD to normalize the values, was used to calculate the utilization of carbon sources^63^. Indices of the functional diversity (Shannon and McIntosh) were calculated according to Gomez *et al*.^64^.

### Genetic analysis of bacterial community

For each soil sample, the DNA was extracted using a Power Soil DNA Isolation Kit (MoBio Laboratories, Inc.) according to the manufacturer’s instructions. Briefly, 0.25 g of soil was loaded into bead tubes containing solution C1, incubated for 10 min at 60 °C and then vortexed for 10 min at maximum speed with the MoBio vortex adapter. The manufacturer’s protocol was followed from this point onward. Following elution, DNA samples concentrated by ethanol precipitation and resuspended in 10 mM Tris. The extracts were assessed for quality and quantity using a spectrophotometer and checked for integrity by 0.8% agarose gel electrophoresis. The V4 regions of the bacterial 16 S rRNA genes were amplified using primers 520 F (5-AYT GGG YDT AAA GNG-3) and 802R (5-TAC NVG GGT ATC TAA TCC-3). After quantified using Quant-iT PicoGreen dsDNA Assay Kit, the PCR products were mixed and sequenced using the 454 GS-FLX Titanium system at Majorbio Bio-Pharm Technology Co., Ltd., Shanghai, China.

The raw sequence reads were denoised using a standard QIIME (Quantitative Insights Into Microbial Ecology)-based pipeline^65^ with default quality settings. They were filtered if they had an average quality score lower than 25, contained fewer than 150 bases and had ambiguous bases. Subsequently, chimeras were checked and eliminated by the MOTHUR program with the UCHIME algorithm^66, 67^. Then, the high-quality sequences were clustered into OTUs at 97% identity using the UCLUST algorithm. The consensus sequence of sequences in each OUT was used as a representative sequence. The taxonomic identity of each representative sequence was assigned to bacteria by using the RDP classifier^68^ trained Greengenes reference database.

We obtained 88 052 high-quality bacterial 16S V_4_ sequences from each soil sample. Based on 97% sequence similarity, 4 133 bacterial OTUs were identified, with a median of 3 399 OTUs in FWS and 3 612 OTUs in AWS (Table 1). Overall, these OTUs belonged to 40 phyla, 125 classes, 232 orders, 365 families and 503 genera. Rarefaction curves in each soil (not shown) indicated that, even at 88 052 reads, we were not capturing the entire community in either soil. Consequently, the total number of OTUs we reported here may be lower than that found in some reports aimed at finding the microbial diversity in other soil types. The indices of bacterial community richness (Chao 1, ACE) and diversity (Shannon and Simpson) were calculated using MOTHUR^66^.

### Data analysis

A paired *t-* test for two independent samples was performed to detect the significant difference using the SPSS 18.0. Heatmap was generated by R packages to exhibit the 31 carbon substrates utilization. PCA was used to identify the microbial characteristics of each sample. Multivariate tests were carried out using PERMANOVA on Euclidean distances with the Adonis command in the vegan package of R. RDA was conducted for analyzing the correlations of microbial characteristics and soil chemical variables. Before the RDA, we conducted selection of the soil variables using the stepwise regression method and the Monte Carlo Permutation test. PCA and RDA analyses were conducted using Canoco 5.0.
